# Supplementary material for: Causal Relationship between Aging and Anorexia Nervosa: A White-Matter-Microstructure-Mediated Mendelian Randomization Analysis
Source: Biomedicines. 2024 Aug 16;12(8):1874. doi: 10.3390/biomedicines12081874 (PMC11351342; doi:10.3390/biomedicines12081874)
Supplement: Supplementary file 1 [file biomedicines-12-01874-s001.zip › Supplementary Figure/Figure S4/Singe SNP plot of MR analysis for TL to ubm-b-1717.pdf]

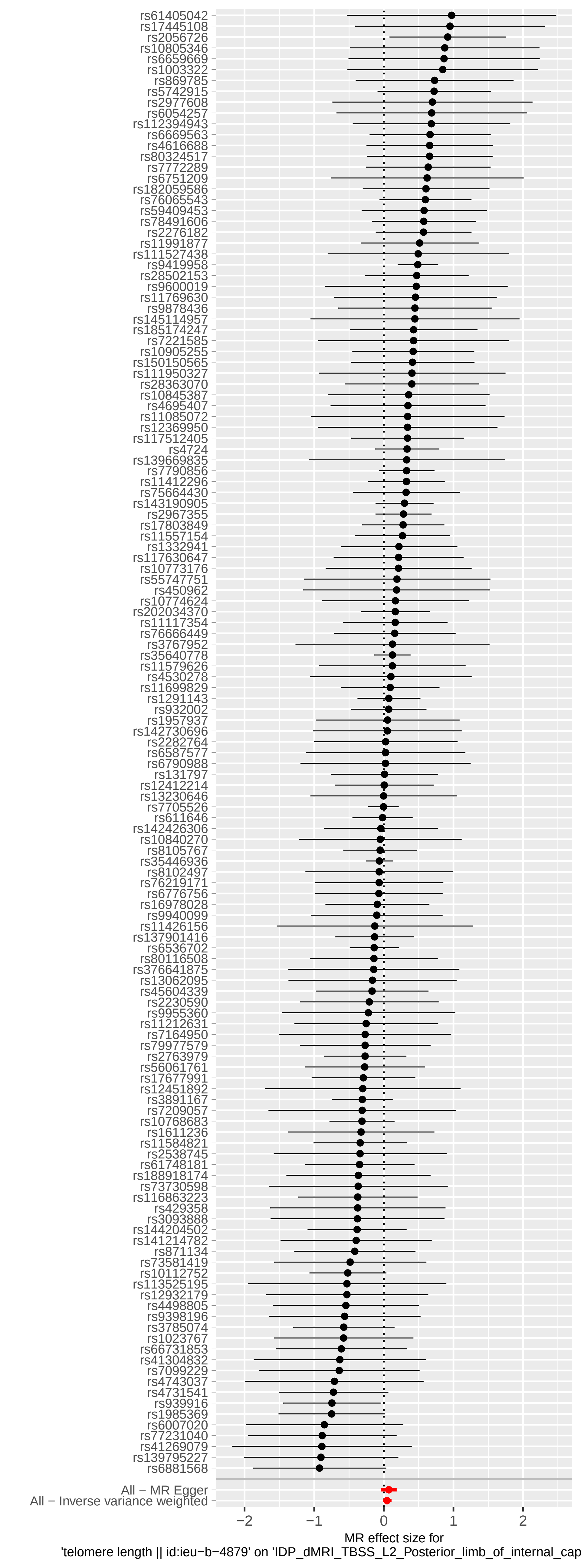
rs61405042
rs17445108
rs2056726
rs10805346
rs6659669
rs1003322
rs869785
rs5742915
rs2977608
rs6054257
rs112394943
rs6669563
rs4616688
rs80324517
rs7772289
rs6751209
rs182059586
rs76065543
rs59409453
rs78491606
rs2276182
rs11991877
rs111527438
rs9419958
rs28502153
rs9600019
rs11769630
rs9878436
rs145114957
rs185174247
rs7221585
rs10905255
rs150150565
rs111950327
rs28363070
rs10845387
rs4695407
rs11085072
rs12369950
rs117512405
rs4724
rs139669835
rs7790856
rs11412296
rs75664430
rs143190905
rs2967355
rs17803849
rs11557154
rs1332941
rs117630647
rs10773176
rs55747751
rs450962
rs10774624
rs202034370
rs11117354
rs76666449
rs3767952
rs35640778
rs11579626
rs4530278
rs11699829
rs1291143
rs932002
rs1957937
rs142730696
rs2282764
rs6587577
rs6790988
rs131797
rs12412214
rs13230646
rs7705526
rs611646
rs142426306
rs10840270
rs8105767
rs35446936
rs8102497
rs76219171
rs6776756
rs16978028
rs9940099
rs11426156
rs137901416
rs6536702
rs80116508
rs376641875
rs13062095
rs45604339
rs2230590
rs9955360
rs11212631
rs7164950
rs79977579
rs2763979
rs56061761
rs17677991
rs12451892
rs3891167
rs7209057
rs10768683
rs1611236
rs11584821
rs2538745
rs61748181
rs188918174
rs73730598
rs116863223
rs429358
rs3093888
rs144204502
rs141214782
rs871134
rs73581419
rs10112752
rs113525195
rs12932179
rs4498805
rs9398196
rs3785074
rs1023767
rs66731853
rs41304832
rs7099229
rs4743037
rs4731541
rs939916
rs1985369
rs6007020
rs77231040
rs41269079
rs139795227
rs6881568
All − MR Egger
All − Inverse variance weighted
−2
−1
0
1
2
MR effect size for
'telomere length || id:ieu−b−4879' on 'IDP_dMRI_TBSS_L2_Posterior_limb_of_internal_cap
